# Mechanistic Insights into Hf(IV)/Zr(IV) Extraction by D2EHPA in Sulfate Media: A DFT Study Supported by Experiments with Industrial Sulfate Liquors

**DOI:** 10.1021/acsomega.6c06044

**Published:** 2026-08-19

**Authors:** Pedro Augusto de Andrade Novaes, Raquel Maia Mingote, Carlos Antonio Morais, Alexandre Carvalho Bertoli, Hélio Anderson Duarte

**Affiliations:** † Grupo de Pesquisa em Química Inorgânica Teórica − GPQIT, Departamento de Química − ICEx, 28114 Universidade Federal de Minas Gerais − UFMG, 31270-901 Belo Horizonte, MG, Brazil; ‡ 54530 Centro de Desenvolvimento da Tecnologia Nuclear − CDTN, 31270-901 Belo Horizonte, MG, Brazil; § Departamento de Química Inorgânica, Instituto de Química, 28125 Universidade Federal do Rio de Janeiro − UFRJ, 21941-901 Rio de Janeiro, RJ, Brazil

## Abstract

The separation of zirconium and hafnium remains a major challenge in hydrometallurgical processing due to their similar chemical behavior and strong tendency toward hydrolysis and complexation in acidic media. In this work, a combined experimental and density functional theory (DFT) investigation was performed to elucidate the mechanism of Hf­(IV)/Zr­(IV) extraction by di­(2-ethylhexyl) phosphoric acid (D2EHPA) in sulfuric acid solutions. DFT calculations indicate that both metals preferentially adopt 8-fold coordination in aqueous solution and undergo favorable first-stage hydrolysis even under strongly acidic conditions. Sulfate coordination was found to be highly exergonic, with bidentate sulfate complexes slightly more stable than monodentate species. Thermodynamic analysis demonstrates that extraction pathways involving concomitant sulfate transfer and proton exchange are significantly more favorable than purely hydroxide-based mechanisms, supporting the formation of neutral sulfate-containing metal–phosphonate complexes in the organic phase. The calculations further indicate a small energetic preference for Hf­(IV) extraction relative to Zr­(IV), consistent with the experimentally observed selectivity of D2EHPA in sulfuric media. Solvent extraction experiments were carried out using sulfate liquors produced directly from zircon concentrate supplied by Indústrias Nucleares do Brasil, reproducing realistic industrial conditions. Aging of the feed solution promoted increased Zr extraction and pronounced sulfate coextraction, indicating substantial changes in aqueous speciation over time. Addition of iodide reduced sulfur coextraction while maintaining relatively high metal extraction efficiencies, suggesting modification of the aqueous coordination environment. The combined theoretical and experimental results demonstrate that Hf/Zr separation in sulfate media is governed by tightly coupled hydrolysis, sulfate coordination, and proton-transfer equilibria, highlighting the critical role of sulfate-containing species in controlling extraction efficiency and selectivity under industrially relevant conditions. From a process-design perspective, the results suggest that tailored extractants capable of modulating the aqueous hydrolysis of Zr­(IV) and Hf­(IV), while preserving efficient coordination in the organic phase, may provide an effective strategy for enhancing extraction selectivity and developing more sustainable Hf/Zr separation processes.

## Graphical abstract

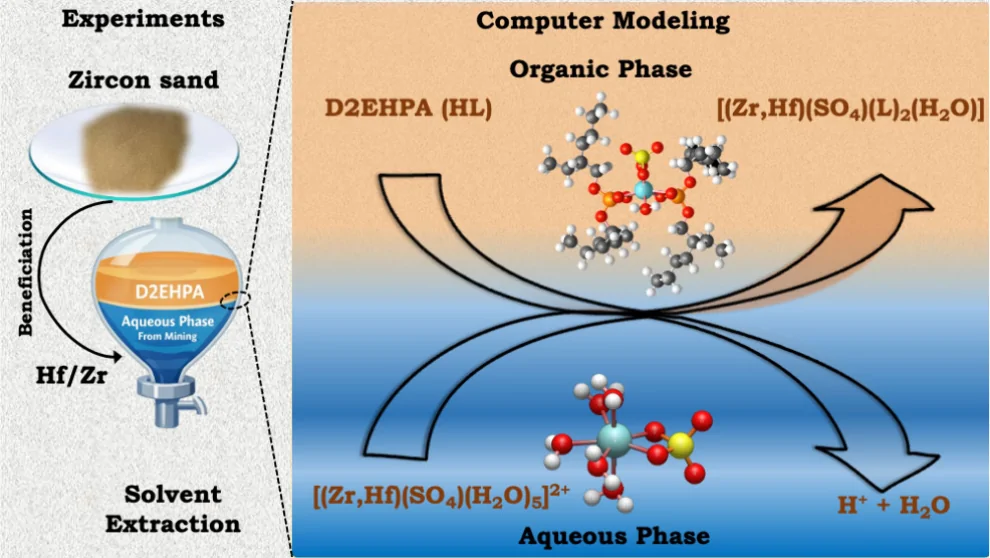

## 1 Introduction

Nuclear-grade zirconium, typically required to achieve a purity of 99.99%, is difficult to obtain due to its natural co-occurrence with hafnium in zirconium minerals. Hafnium is undesirable in nuclear applications because of its exceptionally high thermal neutron absorption cross-section. However, the separation of zirconium and hafnium remains highly challenging owing to their nearly identical chemical properties, including similar ionic radii, charge, and coordination chemistry. Consequently, several separation strategies have been developed, such as solvent extraction, fractional crystallization, halide distillation, and ion exchange.^1,2^

Among the established methods, solvent extraction using methyl isobutyl ketone (MIBK) or tributyl phosphate (TBP) remains the most widely employed at the industrial scale. Current research on zirconium–hafnium separation emphasizes extraction systems that selectively transfer hafnium from zirconium-bearing aqueous phases to the organic phase, exploiting hafnium’s slightly stronger complexation behavior under acidic conditions. The industrial process is typically conducted in highly acidic media (e.g., nitric, hydrochloric, or sulfuric acid) and at elevated ionic strength. Both zirconium and hafnium exist as tetravalent hard Lewis acids (Zr­(IV) and Hf­(IV)), display pronounced oxophilicity, and form stable complexes with oxygen-donor ligands.^1,3,4^

Solvent extraction has been extensively investigated to elucidate the underlying extraction mechanisms and to identify the species involved in both aqueous and organic phases. At the aqueous/organic interface using acid extractants, metal transfer is typically coupled to proton exchange, ensuring that the extracted complex remains electrically neutral in the organic phase. He et al.^5^ investigated the mechanism of Hf/Zr separation from HNO_3_ media using Cyanex 572 (bis­(2,4,4-trimethylpentyl) phosphonic acid) as the extractant, employing ^31^P and ^1^H NMR spectroscopy in combination with FT-IR analysis. Their results demonstrated that Cyanex 572 undergoes deprotonation upon coordination to Zr­(IV) or Hf­(IV), while nitrate ions participate in complex formation, stabilizing the neutral species transferred to the organic phase. At elevated nitrate concentrations (above 2 mol L^–1^), significant nitrate coordination to the metal ions is expected in the aqueous phase.

Di­(2-ethylhexyl) phosphoric acid (D2EHPA) has been identified as promising extractant for Hf/Zr separation, particularly in sulfuric acid media, where it readily forms stable metal–phosphonate complexes with Zr­(IV) and Hf­(IV).^6–9^ It has been proposed that Hf­(IV) exhibits a lower tendency toward hydrolytic polymerization and more readily dissociates from aqueous complexes, thereby facilitating its preferential transfer to the organic phase.

In chloride-, nitrate-, and sulfate-based systems, the nature of the counterion plays a crucial role in determining metal speciation and coordination equilibria in both aqueous and organic phases.^3,4^ The counterion may participate directly in inner-sphere coordination or influence extraction indirectly through changes in ionic strength and complex stability.

More recently, Bertoli et al.^10^ demonstrated that Zr­(IV) and Hf­(IV) exhibit distinct speciation trends in fluoride–nitric acid media, based on a combined experimental–theoretical approach employing paper spray ionization mass spectrometry and density functional theory (DFT) calculations. Wang et al.^11^ also investigated the interaction between the -POOH functional group with Zr­(IV) and Hf­(IV) ions in H_2_SO_4_ medium, providing molecular-level support for the experimentally observed Hf/Zr separation using a functionalized resin.

Most reported studies have been conducted using solutions prepared from analytical-grade chemical compounds.^6–8,12–14^ Moreover, these investigations typically employ simplified binary Hf/Zr systems, which do not adequate reflect the complexity of real industrial processes. In general, it is accepted that metal transfer across the phase boundary is coupled to proton exchange and, in many cases, to the concomitant participation of counterions to ensure the formation of electrically neutral complexes in the organic phase. Recently, Dong et al.^15^ reviewed hafnium/zirconium separation processes employing various extractants and emphasized that, since hafnium represents only 1–3 wt % of the total metal content, its selective extraction is industrially advantageous. They further highlighted that the extraction mechanism is governed by delicate and interdependent equilibria that can be readily perturbed by changes in composition. Therefore, under realistic mining and processing conditions, the separation of zirconium/hafnium becomes even more challenging due to the unavoidable presence of competing metal ions and coordinating anions.

A detailed thermodynamic analysis based on density functional theory (DFT) calculations was performed to interpret the available experimental results and to provide molecular-level insight into the separation mechanism. We propose that, in sulfuric acid medium, the extraction of Hf­(IV)/Zr­(IV) by D2EHPA proceeds via the formation of neutral metal–sulfate–phosphonate complexes, involving the concomitant transfer of coordinated sulfate to the organic phase and proton exchange from the organic to the aqueous phase.

In addition, we have evaluated the efficiency of D2EHPA using stock solutions derived directly from the sulfuric liquor produced directly via the chemical processing of zircon sand supplied by Indústrias Nucleares do Brasil (INB), thereby more closely reproducing realistic processing conditions.^16^

## 2 Methodology

### 2.1 Computational Details

Density functional theory (DFT) calculations were performed for all species arising from the hydrolysis and sulfate coordination reactions in aqueous medium. All calculations were carried out assuming a singlet electronic ground state using restricted Kohn–Sham DFT. Several initial geometries were considered for each species, and full geometry optimizations were performed to identify the lowest-energy structures and establish chemically meaningful coordination models for the Zr­(IV) and Hf­(IV) complexes.

All geometries were fully optimized using the PBE exchange-correlation (XC) functional,^17^ using PBE0 ^18^ and PBE including Grimmés D3 (PBE-D3) dispersion correction^19^ with def2-TZVP basis sets.^20,21^ The harmonic vibrational frequency calculations were carried out to confirm that a true minimum on the potential energy surface (PES) was found.

For zirconium and hafnium, the inner-shell electrons (28 electrons for Zr­(IV) and 60 electrons for Hf­(IV)) were replaced by scalar relativistic effective core potentials (def2-ECP),^22^ as implemented in the ORCA version 5.0.4.^22,23^

Solvation effects were incorporated using the conductor-like polarizable continuum model (CPCM)^24^ during the geometry optimizations and to evaluate the electronic and harmonic frequency calculations. Dielectric constants corresponding to water (ε = 80.15) were used to model the aqueous phase, whereas hexane (ε = 1.89) was employed to represent the organic phase.

### 2.2 Calculation of Free Energies of Complexation

The Gibbs free energies of complex formation in aqueous solution (Δ*G*_aq_) were computed according to Equation 1:

1 $$\Delta G_{\mathrm{aq}}=\Delta E_{g}+\Delta G_{\mathrm{therm}}+\Delta G_{\mathrm{solv}}$$

Where Δ*E*_g_ corresponds to the electronic energy obtained from gas-phase single-point calculations, and Δ*G*_therm_ represents the thermal correction derived from vibrational frequency analysis within the rigid-rotor-harmonic-oscillator approximation (RRHO) approximation. The solvation contribution (Δ*G*_solv_) was estimated using the CPCM implicit solvation model. Recently, Xinjie Qi et al.,^25^ investigated the mechanism of the Hf/Zr separation by dibenzoylmethane, using similar approach.

For reactions involving proton formation, the experimental solvation free energy of the proton (−264.6 kcal mol^–1^) was adopted.^26^ Thermodynamic treatment follows the experimental proton correction approach reported in the literature.^27–29^

Single point energy calculations were performed using PBE XC with extended def2-TZVPPD basis sets to assess the effect of basis-set enlargement on the calculated reaction energies. Although the absolute reaction free energies differ by up to 10 kcal mol^–1^ from those obtained with the def2-TZVP basis set, these changes are systematic and the relative thermodynamic trends remain unchanged (Tables S5, S7, and S9). In particular, the energetic ordering of the reaction pathways and the conclusions regarding the preferred coordination modes are fully preserved.

### 2.3 Experimental Details

#### 2.3.1 Production of Hf/Zr Sulfate from a Primary Sample of Zirconium Concentrate and Preparation of Solutions

Hafnium/zirconium sulfate was obtained from a sample of zirconium concentrate supplied by Indústrias Nucleares do Brasil (INB), through a multistep procedure comprising: (i) alkaline fusion of the concentrate with commercial NaOH (99% w/w); (ii) washing with water; (iii) acid leaching with sulfuric acid; and (iv) crystallization of resulting Hf/Zr sulfate. The mineralogical characterization of the primary sample is presented in Table S1 (Supporting Information).

The matrix solutions were prepared by dissolving an appropriate amount of the obtained Hf/Zr sulfate in distilled water, followed by adjustment of the free acidity with sulfuric acid. The solutions were allowed to equilibrate for 24 h at room temperature. The final concentrations in the matrix solution were approximately 20.24 g L^–1^ for Zr and 0.43 g L^–1^ for Hf, in a medium with a free acidity of 2.4 mol L^–1^ (H^+^(aq)).

### 2.4 Solvent Extraction and Analytical Procedures

The commercial extractant di­(2-ethylhexyl)­phosphoric acid (D2EHPA, 97% w/w) was supplied by Albright & Wilson Americas Inc. and diluted in kerosene (Exxsol D100, Exxon Chemicals Ltd.) to the 0.06 mol L^–1^. The organic solvent was used without further purification.

Liquid–liquid extraction experiments were carried out at 25 °C using equal volumes of aqueous and organic phases (Vaq/Vorg = 1). In each experiment, 25 mL of aqueous solutions was contacted with 25 mL of organic phase under mechanical stirring. After 30 min contact time, the phases were separated using separatory funnels, and aliquots were collected for analysis.

The solvent extraction tests were identified as follows:

- **1SX**: extraction using zirconium sulfate solution stored for 24 h.
- **2SX**: extraction using zirconium sulfate solution stored for 96 h.
- **3SX**: extraction using zirconium sulfate solution stored for 96 h; iodide was added to form a solution of 0.1 g L^−1^ during Hf/Zr extraction.

The elemental concentrations in the aqueous phase were determined by Inductively Coupled Plasma Optical Emission Spectroscopy (ICP-OES - Spectro Arcos). The analytical Relative Standard Deviation (RSD) was 3.3% for Zr and 2.4% for Hf. The metal concentrations in the organic phase were calculated by mass balance. Sulfur concentrations in the organic phase were determined by Energy-Dispersive X-ray Fluorescence (EDXRF - Thermo Fisher Scientific). Duplicate experiments were conducted, and the errors related to the experimental results were more or less ± 5%.

The percentage extraction (%E) and distribution coefficient (D) were defined as

2 $$\%E=\frac{C_{org}V_{org}}{C_{org}.V_{org}+C_{aq}.V_{aq}}$$

3 $$D=\frac{C_{org}}{C_{aq}}$$

The separation factor *α*_*Hf/Zr*_ was given by

4 $$\alpha_{Hf/Zr}=\frac{D_{Hf}}{D_{Zr}}$$

## 3 Results and Discussion

### 3.1 Aqueous Complexation, Hydrolysis, and Sulfate Coordination of Zr­(IV) and Hf­(IV) Species

Modeling highly charged transition-metal complexes in aqueous solution remains a significant challenge for first-principles calculations, particularly for species in high oxidation states. The coordination number (CN) of water molecules surrounding Zr­(IV) and Hf­(IV) in aqueous media has long been debated,^10,30–32^ with both experimental and computational studies suggesting preferred coordination numbers of 7 or 8.

This ambiguity highlights the intrinsic difficulty of accurately describing the thermodynamics of water coordination around these highly charged metal centers. Such sensitivity is reflected in the Gibbs free energies associated with the stepwise hydration process described in Equation 5. The corresponding results are summarized in Table 1 and further detailed in Figure S1 and Tables S3 and S4.

5 $${\left[M{\left(H_{2}O\right)}_{7}\right]}^{4+}\left(aq\right)+H_{2}O\left(l\right)\to {\left[M{\left(H_{2}O\right)}_{8}\right]}^{4+}\left(aq\right)$$

Hereafter, M denotes either Zr­(IV) or Hf­(IV).

**Table 1:** Reaction Gibbs Free Energies^a^ and Average Hf­(IV)/Zr­(IV)–OH_2_ Bond Distances (Å) for CN = 7–8 Complexes, Obtained from PBE/CPCM Calculations and Compared with EXAFS Data

	PBE			EXAFS^b^	
		Bond Length (Å)			
Metal	ΔG (kcal mol^–1^)	CN = 7	CN = 8	CN = 7	CN = 8
**Zr(IV)**	–10.5	2.17	2.26	2.14	2.19
**Hf(IV)**	–11.2	2.16	2.20	2.13	2.16

a According to Eq. 5.

b Ref.^33^

The values reported in Table 1 indicate that both Zr­(IV) and Hf­(IV) preferentially adopt a coordination number of 8, with the CN = 8 species being at least 10.5 and 11.2 kcal mol^–1^ more stable than the corresponding CN = 7 complexes, respectively, at the PBE/CPCM level. As shown in Table 1, the average metal–oxygen bond distances for the CN = 8 complexes are, as expected, slightly longer and are in good agreement with the experimental data reported by Hagfeldt et al.^33^

The average Zr­(IV)–OH_2_ and Hf­(IV)–OH_2_ bond distances indicate that the ionic radius of Zr­(IV) is only slightly larger than that of Hf­(IV). The smaller ionic radius of Hf­(IV) has been attributed to relativistic effects and to its higher effective nuclear charge, associated with the presence of filled inner 4f^14^ electrons.^33^ These factors may contribute to subtle differences in coordination behavior and complex stability between Zr­(IV) and Hf­(IV).^36^

#### 3.1.1 Hydrolysis of the Zr­(IV) and Hf­(IV)

Zr­(IV) and Hf­(IV) undergo hydrolysis in aqueous solution, leading to the formation of various hydrolyzed species. According to reported p*K*_a_ values, at pH < 1.5, Zr­(IV) and Hf­(IV) are predominant as monomeric aqua complexes, such as [Zr­(H_2_O)_8_]^4+^(aq) and [Hf­(H_2_O)_8_]^4+^(aq).^34–36^ In the pH range 1.5–3.0, monomeric hydrolyzed species, including [Zr­(OH)]^3+^(aq) and [Zr­(OH)_2_]^2+^(aq), (and their Hf­(IV) analogues), become increasingly significant.

Liquid–liquid extraction is typically carried out at high acid concentrations (∼3 mol L^–1^), where proton activity strongly influences hydrolysis equilibria.

Accordingly, we propose a general description of the hydrolysis reactions occurring in the aqueous phase, as represented by Equations 6-([Disp-formula eq8]):

6 $${\left[M{\left(H_{2}O\right)}_{x}\right]}^{4+}\left(aq\right)\to {\left[M\left(OH\right){\left(H_{2}O\right)}_{\left(x-1\right)}\right]}^{3+}\left(aq\right)+H^{+}\left(aq\right)$$

7 $${\left[M\left(OH\right){\left(H_{2}O\right)}_{\left(x-1\right)}\right]}^{3+}\to {\left[M{\left(OH\right)}_{2}{\left(H_{2}O\right)}_{\left(x-2\right)}\right]}^{2+}\left(aq\right)+H^{+}\left(aq\right)$$

8 $${\left[M\left(OH\right){\left(H_{2}O\right)}_{\left(x-1\right)}\right]}^{3+}\to {\left[M\left(O\right){\left(H_{2}O\right)}_{\left(x-1\right)}\right]}^{2+}\left(aq\right)+H^{+}\left(aq\right)$$

Here, *x*(= 7 or 8) represents the number of coordinated water molecules. Equation 6 describes the first hydrolysis step, whereas Equations 7 and ([Disp-formula eq8]) correspond to the second hydrolysis stage, leading to the formation of either dihydroxo or oxo species, respectively.

The hydrolysis reaction free energies of Zr­(IV) and Hf­(IV) were estimated, and the optimized structures, together with a schematic representation of the hydrolysis pathways, are presented in Figure 1.

**Figure 1:**
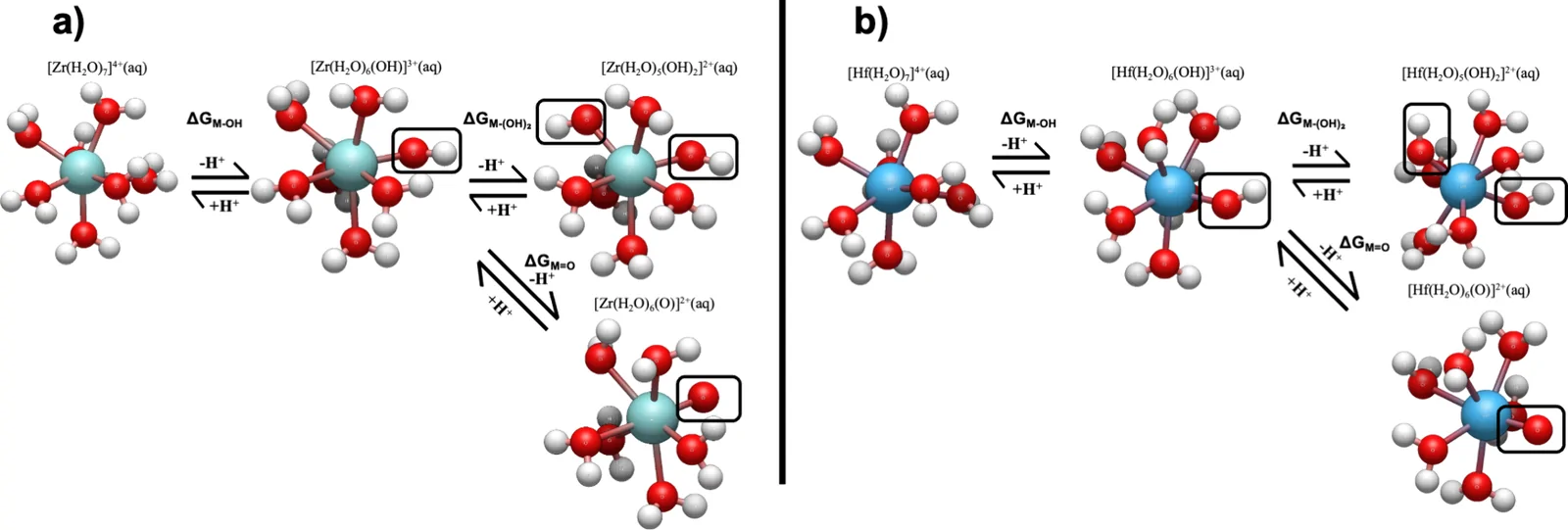
Optimized structures for (a) Zr­(IV) and (b) Hf­(IV) hydrolysis reactions (CN = 7) calculated at the PBE/def2-TZVP level with CPCM.

Similar hydrolysis pathways have been reported in the literature. Previous studies reported that, in 2 mol·L^–1^ perchloric acid at 25 °C, zirconium species in dilute solutions exhibit an average charge between 3+ and 4+, with no evidence of polymerization. This behavior is consistent with the coexistence of M^4+^(aq) and MOH^3+^(aq) species, as well as the possible presence of M­(OH)_2_^2+^(aq) and M­(O)^2+^(aq) species. At higher zirconium concentrations, however, clear evidence of polymerization and more extensive hydrolysis have been observed.^34,35,37,38^

The reaction free energies corresponding to the processes depicted in Figure 1 are reported in Table 2 for coordination numbers (CN) of 7 and 8. The optimized structures for the CN = 8 species, together with the corresponding hydrolysis reaction free energies calculated using the PBE0 and PBE-D3 functionals, are presented in Figure S2 and Table S5.

**Table 2:** Calculated Hydrolysis Reaction Free Energies (kcal mol^–1^) for Coordination Number (CN) = 7 and 8, Obtained at the PBE/CPCM Level

		1st Stage	2nd Stage	
Metal	CN	Δ*G*_M–OH_	Δ*G*_M‑(OH)_2__	Δ*G*_M=O_
**Zr(IV)**	7	–32.6	–12.2	–1.1
	8	–19.7	–4.7	–2.2
**Hf(IV)**	7	–31.1	–7.9	6.5
	8	–17.0	6.5	5.9

The first hydrolysis reaction is thermodynamically favorable, indicating that it may occur even under acidic conditions. For both metals, the CN = 7 is more stable by at least 12 kcal mol^–1^ relative to the corresponding CN = 8 complex. The second hydrolysis step preferentially leads to the formation of the dihydroxo species [M­(OH)_2_]^2+^ rather than the oxo species [MO]^2+^.

### 3.2 Sulfate Coordination of Zr­(IV) and Hf­(IV) in Aqueous Solution

The sulfate ion (SO_4_^2–^) plays a crucial role in coordination with Zr­(IV) and Hf­(IV).^39,40^ According to the literature, sulfate forms stable complexes with both metal ions in aqueous solutions.^1^ High concentrations of Zr­(IV) and Hf­(IV) are typically generated during mineral dissolution processes,^41^ and the presence of zirconium sulfate species in highly acidic aqueous media has been experimentally demonstrated.^39,42,43^

Coordination of sulfate to Zr­(IV) and Hf­(IV) is therefore significant and may strongly influence the chemical speciation of these ions in solution. The general sulfate coordination reactions occurring in the aqueous phase are represented by Equations 9 and ([Disp-formula eq10]):

9 $${\left[M{\left(H_{2}O\right)}_{x}\right]}^{4+}\left(aq\right)+SO_{4}^{2-}\left(aq\right)\to {\left[M\left(SO_{4}\right){\left(H_{2}O\right)}_{x-1}\right]}^{2+}\left(aq\right)+H_{2}O\left(l\right)$$

10 $${\left[M\left(SO_{4}\right){\left(H_{2}O\right)}_{x-1}\right]}^{2+}\left(aq\right)\to {\left[M\left(SO_{4}\right){\left(H_{2}O\right)}_{x-2}\right]}^{2+}\left(aq\right)+H_{2}O\left(l\right)$$

Here, x (= 7 or 8) also represents the number of coordinated water molecules. Equation 9 corresponds to the first coordination step, in which the sulfate ion binds in a monodentate coordination mode, whereas Equation 10 describes the second step, involving bidentate coordination mode. The corresponding optimized structures are illustrated in Figure 2.

**Figure 2:**
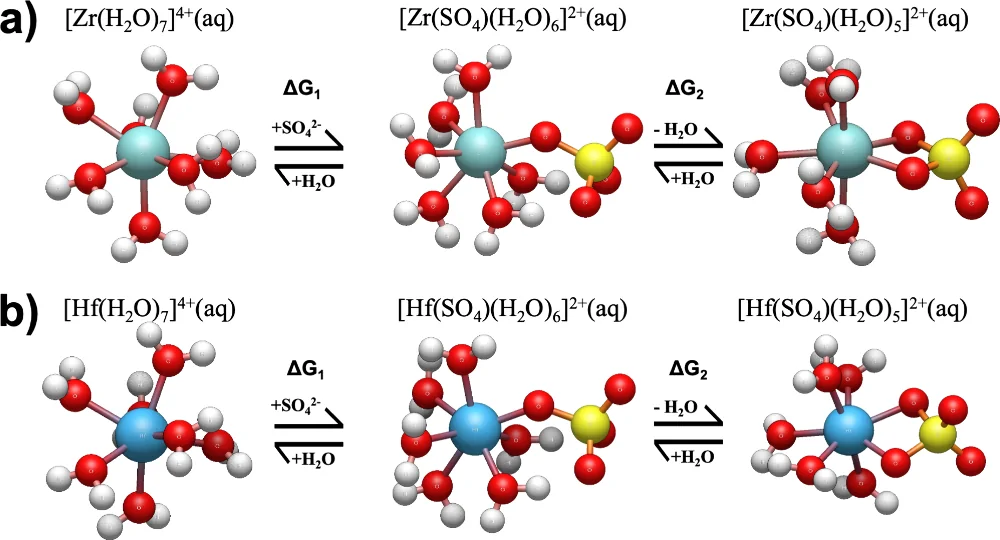
Optimized structures for sulfate coordination to (a) Zr­(IV) and (b) Hf­(IV) complexes with CN = 7 at the PBE level.

The energy values for the reactions depicted in Figure 2 are reported in Table 3. Here, Δ*G*_1_ corresponds to the monodentate coordination of the sulfate ion, whereas Δ*G*_2_ represents the subsequent bidentate coordination step. The reaction free energies were calculated for both seven- and eight-coordinate complexes. Complexation energies obtained using the PBE0 and PBE-D3 XC functionals follow the same trends and are provided in the Supporting Information.

**Table 3:** Calculated Complexation Free Energies (kcal mol^–1^) for Sulfate Coordination Reactions

	PBE/CPCM (kcal mol^–1^)					
	CN = 7			CN = 8		
Metal	Δ*G*_1_	Δ*G*_2_	Δ*G*_1_ + Δ*G*_2_	Δ*G*_1_	Δ*G*_2_	Δ*G*_1_ + Δ*G*_2_
**Zr(IV)**	–67.7	–1.9	–69.6	–55.3	–4.5	–59.8
**Hf(IV)**	–67.2	–3.8	–71.0	–53.7	–3.5	–57.2

For both metals, the most stable complexes have CN = 7, whereas the corresponding CN = 8 complexes are at least 10 kcal mol^–1^ higher in free energy. In addition, bidentate sulfate coordination is favored by at least 2 kcal mol^–1^ over the monodentate mode, consistent with the chelate effect. Since Zr­(IV) and Hf­(IV) are hard Lewis acids, their interaction with the hard Lewis base sulfate ion is predominantly ionic in character. The preference for the CN = 7 complexes reflects the balance between the strong electrostatic interaction with the bidentate sulfate ligand and the increased ligand–ligand repulsion within the first coordination sphere. Because the M–O­(sulfate) bonds are shorter than the M–OH_2_ bonds (Table S22 and S23), coordination of sulfate draws the surrounding ligands closer together, making the addition of an eighth ligand less favorable. Furthermore, the slightly smaller ionic radius of Hf­(IV) results in a slightly stronger electrostatic interaction with sulfate, leading to an additional stabilization of approximately 1.4 kcal mol^–1^ relative to the corresponding Zr­(IV) complexes.

In highly acidic solutions, sulfate-containing species are also present. Previous studies by Ryabchikov et al.^34^ reported the formation of ZrSO_4_^2+^(aq) and HfSO_4_^2+^(aq) complexes in agreement with the energetic trends obtained in our calculations. The additional stability conferred by sulfate coordination may also influence the structural stability of the complexes in the organic phase, favoring configurations that retain coordinated sulfate. This effect is therefore essential for understanding the mechanism of liquid–liquid extraction.

### 3.3 D2EHPA Coordination with Zr­(IV) and Hf­(IV) in the Organic Phase

The ground-state structures of the D2EHPA (HL) ligand and its hydrogen-bonded dimer were optimized, as shown in Figure 3. All optimized geometries exhibit only real vibrational frequencies, confirming that they correspond to true minima on the potential energy surface (PES). The binding energy of the hydrogen-bonded D2EHPA dimer is calculated to be – 20.8 kcal mol^–1^ (see Table S11).

**Figure 3:**
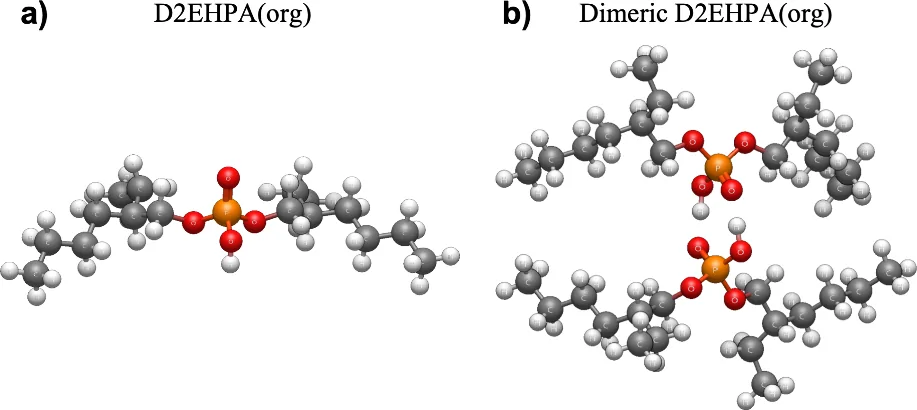
Optimized structures in PBE with CPCM of (a) D2EHPA and (b) Dimeric D2EHPA.

To model coordination in the organic phase, we adopt a chemical framework in which all extracted species are electrically neutral overall. Under this assumption, four possible neutral complexes are proposed, arising from combinations of deprotonated D2EHPA (L^–^), sulfate, hydroxide, and water ligands:

(I) [M­(L)_2_(SO_4_)­(H_2_O)_*x*_]­(org)
(II) [M­(L)­(OH)­(SO_4_)­(H_2_O)_*x*_]­(org)
(III) [M­(L)_2_(OH)_2_(H_2_O)_*x*_]­(org)
(IV) [M­(L)­(OH)_3_(H_2_O)_*x*_]­(org)

Here, x (= 7 or 8) represents the number of coordinated water molecules consistent with the adopted coordination number. The formation of neutral complexes in the organic phase is consistent with the experimental evidence reported by Lee et al.,^7^ who demonstrated that neutral species predominate during solvent extraction with D2EHPA.

Moreover, the presence of sulfate ions plays a significant role in the extraction mechanism. Sulfate coordination can stabilize metal–ligand structures in the organic phase, thereby influencing both extraction efficiency and selectivity. As observed experimentally, the extraction percentage decreases with increasing sulfuric acid concentration, suggesting that the sulfate environment affects the formation and stability of neutral complexes. This behavior is therefore a key factor in understanding the liquid–liquid extraction mechanism of Zr­(IV) and Hf­(IV) in sulfate media.

#### 3.3.1 Liquid–Liquid Extraction Mechanism of Zr­(IV) and Hf­(IV) with D2EHPA

The extraction mechanism of Zr­(IV) and Hf­(IV) by D2EHPA (HL) in the organic phase was investigated to elucidate the influence of sulfate coordination and hydrolysis on complex stability. The general extraction reactions are described by Equations 11 – [Disp-formula eq14]:

I)
11 $$\begin{array}{l} {\left[M\left(SO_{4}\right){\left(H_{2}O\right)}_{x}\right]}^{2+}\left(aq\right)+2HL\left(org\right)\to \left[M{\left(L\right)}_{2}\left(SO_{4}\right){\left(H_{2}O\right)}_{\left(x-4\right)}\right]\left(org\right)+\\ 4H_{2}O\left(l\right)+2H^{+}\left(aq\right) \end{array}$$
II)
12 $$\begin{array}{l} {\left[M\left(SO_{4}\right){\left(H_{2}O\right)}_{x}\right]}^{2+}\left(aq\right)+HL\left(org\right)\to \left[M\left(L\right)\left(OH\right)\left(SO_{4}\right){\left(H_{2}O\right)}_{\left(x-2\right)}\right]\left(org\right)+\\ 2H_{2}O\left(l\right)+2H^{+}\left(aq\right) \end{array}$$
III)
13 $$\begin{array}{l} {\left[M{\left(OH\right)}_{2}{\left(H_{2}O\right)}_{x}\right]}^{2+}\left(aq\right)+2HL\left(org\right)\to \left[M{\left(L\right)}_{2}{\left(OH\right)}_{2}{\left(H_{2}O\right)}_{\left(x-4\right)}\right]\left(org\right)+\\ 4H_{2}O\left(l\right)+2H^{+}\left(aq\right) \end{array}$$
IV)
14 $$\begin{array}{l} {}[M{\left(OH\right)}_{2}{\left(H_{2}O\right)}_{x}{]}^{2+}\left(aq\right)+HL\left(org\right)\to \left[M\left(L\right){\left(OH\right)}_{3}{\left(H_{2}O\right)}_{x-2}\right]\left(org\right)+\\ 2H_{2}O\left(l\right)+2H^{+}\left(aq\right) \end{array}$$

Here, L represents the deprotonated D2EHPA ligand, and x (=5 or 6) corresponds to the number of coordinated water molecules in the aqueous species. The proposed organic-phase species were modeled as neutral complexes, consistent with the charge balance required for solubility in the organic phase.^44^

Figure 4 illustrates the reaction pathway of the liquid–liquid extraction process at the aqueous–organic interface for CN = 7, as modeled at the PBE/CPCM level of theory.

**Figure 4:**
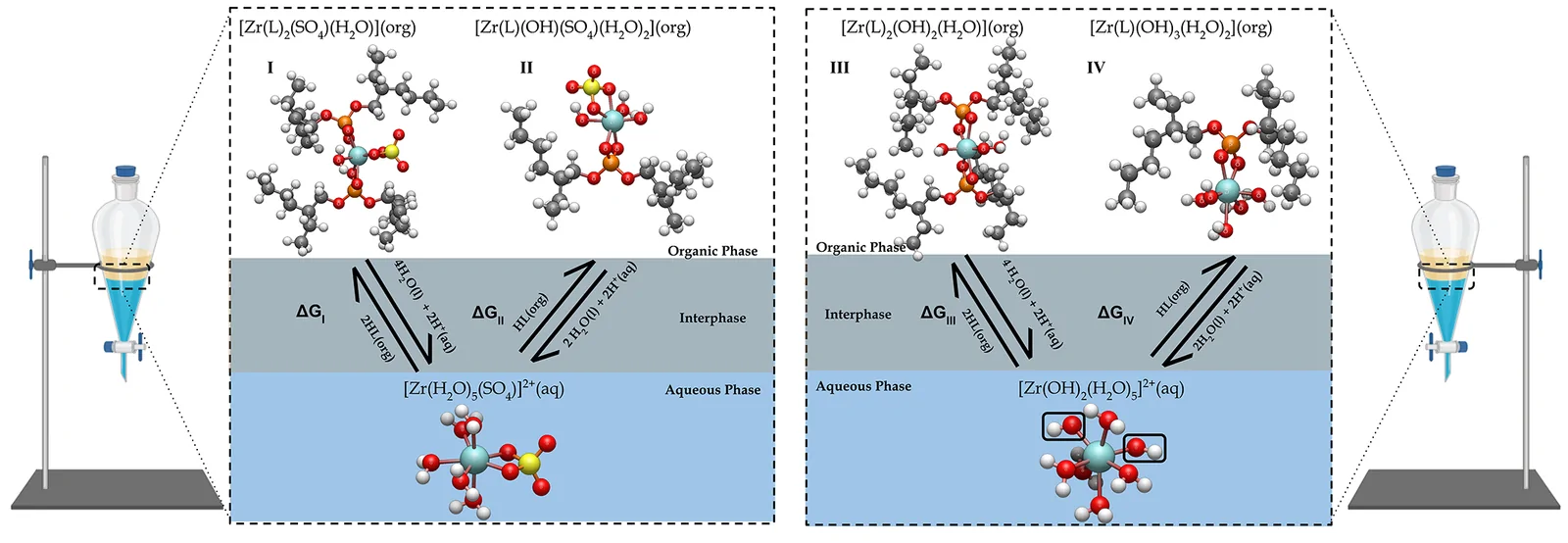
Reaction pathway for the liquid–liquid extraction of Zr­(IV) at the aqueous/organic interface (CN = 7), computed at the PBE/CPCM level of theory.

The energy values for the liquid–liquid extraction reactions depicted in Figure 4 are reported in Table 4 for D2EHPA-sulfate coordination pathways. Reaction free energies calculated using the PBE0 and PBE-D3 XC functionals are provided in the Supporting Information.

**Table 4:** Calculated Reaction Free Energies (kcal mol^–1^) for the Liquid–Liquid Extraction Pathways at the Aqueous-Organic Interface^a^

M	Zr(IV)		Hf(IV)	
CN	7	8	7	8
**Δ** *G*_**I**_	–17.9	–13.3	–18.4	–11.5
**Δ** *G*_**II**_	–16.6	–6.8	–10.4	–4.9
**Δ** *G*_**III**_	–7.2	–9.1	–10.3	–7.3
**Δ** *G*_**IV**_	1.0	5.9	2.8	5.6

a Computed at the PBE/CPCM level of theory.

The results in Table 4 show that Δ*G*_I_ < Δ*G*_II_ < Δ*G*_III_ ≪ Δ*G*_IV_, highlighting the crucial role of sulfate in the extraction mechanism. Sulfate coordination contributes significantly to the stabilization of the organic-phase complexes. This stabilization is reflected in the calculated free energies: Δ*G*_I_ and Δ*G*_II_, which correspond to pathways involving sulfate transfer from the aqueous to the organic phase and exhibit more favorable (lower) values.

These findings are consistent with experimental observations reported by Banda et al.,^44^ who showed that D2EHPA achieves higher extraction efficiency and selectivity for Hf­(IV) over Zr­(IV) in sulfuric acid media. Moreover, Δ*G*_*I*_ is consistently the most favorable pathway and is slightly more stable for Hf­(IV) than for Zr­(IV) across the different XC functionals employed (see Supporting Information).

The enhanced stability of sulfate-containing complexes in the organic phase suggests that the sulfate anion plays a key role in the extraction mechanism, influencing both selectivity and complex geometry. This observation is consistent with previous reports indicating that the extraction efficiency of D2EHPA increases with increasing sulfuric acid concentration.^8^

Finally, although Δ*G*_IV_ corresponds to the least favorable process among the modeled reactions, its high degree of hydroxylation (three OH groups) suggests that such species are unlikely to form under strongly acidic conditions, where Zr­(IV) and Hf­(IV) are expected to remain predominantly as monomeric species and extensive hydroxylation and polymerization are suppressed.^34,35,37,38^

The computational results support a mechanism in which extraction proceeds predominantly through the formation of neutral sulfate-containing complexes stabilized by D2EHPA in the organic phase. The calculated free energy trends indicate that pathways involving sulfate coordination and concomitant proton release (Δ*G*_I_ and Δ*G*_II_) are thermodynamically favored, in agreement with the experimentally observed sulfur coextraction. Sulfate thus plays a dual role: it stabilizes the metal center through inner-sphere coordination and facilitates charge neutralization during transfer across the aqueous–organic interface. The slightly greater stabilization calculated for Hf­(IV) relative to Zr­(IV), particularly for pathway I, provides a thermodynamic basis for the experimentally observed selectivity of D2EHPA in sulfuric acid media. Overall, the extraction process can be described as a coupled ligand-exchange and proton-transfer mechanism strongly modulated by sulfate coordination equilibria in the aqueous phase.

One could argue that the kinetics of the extraction process at the aqueous/organic interface may also contribute to the Hf/Zr separation selectivity. However, determining the reaction pathways for metal coordination coupled with proton transfer in solution is a highly challenging problem because of the large number of possible reaction channels and solvent configurations that must be sampled. In the present system, this complexity is further increased by the extraction occurring at the aqueous/organic interface, where the organization and dynamics of both solvent phases must be explicitly considered. Consequently, a reliable description of the activation barriers would require extensive sampling using advanced computational approaches, such as molecular dynamics combined with free-energy calculations, which are beyond the scope of the present work and the capabilities of conventional static DFT calculations. Therefore, the present study is focused on the thermodynamic aspects of the extraction mechanism, which provide the molecular basis for understanding the experimentally observed trends.

#### 3.3.2 Experimental Evidence of Extraction of Hf­(IV) and Zr­(IV) Solutions with D2EHPA

Liquid–liquid solvent extraction experiments using D2EHPA and stock solutions derived directly from mineral sources provide experimental evidence of how ionic strength and the presence of potentially competing impurity ions affect the extraction process.

The extraction results summarized in Table 5 show consistent differences among the three experimental conditions evaluated. The composition of the sulfuric acid feed liquor is shown at Table S2, indicating the presence of Al^3+^, Fe^3+^, and silicates.

**Table 5:** Elemental Concentrations of Zr, Hf, and S in the System after the Liquid–Liquid Extraction^a^

	Sulfuric acid feed liquor^b^		Aqueous Phase		Organic Phase		
Solvent Extraction Tests	Zr	Hf	Zr	Hf	Zr	Hf	S^c^
**1SX**	20238 (221.9)	428 (2.4)	18928 (207.5)	354 (1.98)	1311 (14.4)	74 (0.41)	271 (8.45)
**2SX**			17813 (195.3)	354 (1.98)	2426 (26.6)	74 (0.41)	669 (20.9)
3SX + I			18111 (198.5)	366 (2.05)	2127 (23.3)	62 (0.35)	326 (10.2)

a Values in mg L^–1^ and, in parentheses, in 10^–3^ mol L^–1^.

b Total sulfate content is 1.4 mol L^–1^.

c The sulfur content in a blank experiment, performed under the same aqueous-phase acidity conditions, was 55 mg L^–1^ (1.71 × 10^–3^ mol L^–1^).

The Zr concentration in the organic phase increased for samples **2SX** and **3SX** relative to **1SX**. In parallel, sulfur coextraction was particularly pronounced for the aged solution **2SX** (669 mg L^–1^), corresponding to an increase of approximately 147% compared to **1SX** (271 mg L^–1^). This substantial rise in sulfur content strongly suggests enhanced transfer of sulfate-coordinated metal species to the organic phase after solution aging.

Storage of the aqueous metal solution for 96 h likely altered the speciation of zirconium and hafnium in acidic sulfate media. Under such conditions, Zr^4+^ and Hf^4+^ may undergo hydrolysis and sulfate coordination, leading to the formation of oxo/hydroxide and sulfate-containing complexes or aggregates.^45,46^ These species may exhibit greater affinity for organophosphorus such as D2EHPA, thereby favoring extraction. The increased sulfur concentration detected in the organic phase supports the hypothesis of coextraction of sulfate-containing Hf­(IV)/Zr­(IV) species.^14,15,47^

A patent by Lee et al.^48^ reported the use of iodide as an additive in D2EHPA-based Hf­(IV)/Zr­(IV) separation with D2EHPA in sulfuric media. Accordingly, iodide was introduced in the present study to evaluate its effect under realistic feed conditions. The addition of iodide during extraction (**3SX**) reduced sulfur coextraction (326 mg L^–1^) relative to the aged solution without iodide (**2SX**), while maintaining higher Zr extraction than the observed for **1SX**. These results suggest that iodide modifies the aqueous coordination environment of the metal ions, likely by competing with sulfate and partially displacing it from the inner coordination sphere. Such effects may alter the stability and stoichiometry of the extracted neutral complexes, reducing sulfate transfer to the organic phase without suppressing metal extraction. However, further studies are required to clarify the mechanistic role of iodide in the extraction process.

Table 6 summarizes the percentage extraction (%E), distribution coefficients (D), and separation factors (*α*_*Hf/Zr*_) obtained under the different experimental conditions. The freshly prepared solution (**1SX**) exhibited the highest separation factor (*α*_*Hf/Zr*_ = 3.03), with 6.5% extraction for Zr and 17.3% for Hf, confirming preferential extraction of Hf­(IV) under these conditions.

**Table 6:** Percentage Extraction (%E), Distribution Coefficient (D), and Separation Factor (α_Hf/Zr_) for the Hf/Zr System

	Zr		Hf		
Samples in the organic phase	%E	D	%E	D	α_Hf/Zr_
**1SX**	6.5	0.069	17.3	0.209	3.03
**2SX**	12.0	0.136	17.3	0.209	1.53
3SX+I	10.5	0.117	14.5	0.169	1.44

Upon storage of the feed solution (**2SX**), Zr extraction increased significantly to 12.0%, while Hf extraction remained essentially unchanged (17.3%). As a consequence, the separation factor decreased to 1.53. This behavior suggests that aging of the sulfate feed promotes the formation of more extractable Zr-containing species, likely involving hydrolyzed and/or sulfate-coordinated complexes, thereby reducing selectivity.

The addition of iodide (**3SX**) resulted in intermediate behavior: Zr extraction (10.5%) remained higher than in the freshly prepared solution but lower than in the aged solution without iodide, while Hf extraction slightly decreased (14.5%). Consequently, the separation factor further decreased to 1.44. These results indicate that iodide alters the aqueous coordination equilibria, influencing both extraction efficiency and selectivity.

Higher separation factors (>30) have been reported in the literature;^1,6,12,13^ ^,^ ^15,45,49^ however, such studies typically employ analytical-grade reagents, simplified binary systems, and controlled acid compositions. Under those optimized laboratory conditions, the speciation is considerably less complex than in real feed solutions derived from mineral concentrates. Banda et al.^44^ investigated the D2EHPA-based Hf­(IV)/Zr­(IV) separation in sulfuric media and observed that increasing the D2EHPA concentration reduced the separation factor from 10.6 to 1.5 in 1 mol L^–1^ H_2_SO_4_, demonstrating the strong sensitivity of selectivity to extraction conditions.

Although acidity conditions employed in the present work are within the same general range as those reported in the literature, the optimal Hf­(IV)/Zr­(IV) separation strongly depends on the characteristics of the feed solution and the origin of the metal source. The mineral source, impurity profile, and beneficiation processes involved in the early stages of sulfate production significantly influence metal speciation and, consequently, extraction performance.^7^ Therefore, efficient separation strategies must be specifically tailored to the characteristics of the industrial feedstock rather than relying solely on results obtained from idealized analytical systems.

## 4 Conclusion

A detailed thermodynamic mechanism for the separation of Hf­(IV)/Zr­(IV) by D2EHPA in sulfuric acid medium was proposed based on density functional theory (DFT) calculations. The results indicate that both metals preferentially adopt 8-fold coordination in aqueous solution and undergo favorable first-stage hydrolysis even under highly acidic conditions. Sulfate coordination is strongly exergonic and stabilizes hydrolyzed species, with bidentate binding being slightly preferred. Extraction pathways involving concomitant sulfate transfer and proton exchange are thermodynamically favored over purely hydroxide-based pathways, supporting a mechanism in which neutral sulfate-containing metal–phosphonate complexes are formed in the organic phase.

Partially hydrolyzed species, either containing or lacking coordinated sulfate, are also thermodynamically accessible; however, they are predicted to be at least 6 kcal mol^–1^ less stable than the corresponding sulfate-containing complexes. This energetic trend is consistent with experimental observations^12^ showing that higher acidity enhances Zr­(IV) and Hf­(IV) extraction, since strongly acidic conditions favor sulfate-coordinated species over extensively hydrolyzed forms. A consistent, although small, energetic preference for Hf­(IV) relative to Zr­(IV) was calculated for the most favorable extraction pathway; however, this difference remains within the intrinsic uncertainty of the DFT level of theory employed. Nevertheless, the results clearly demonstrate that sulfate exerts a dual role by controlling aqueous speciation and directly stabilizing the extracted neutral complexes. In particular, partially hydrolyzed sulfate-containing species favor the Zr­(IV) extraction pathway (Δ*G*_II_), helping to explain the experimentally observed decrease in the separation factor with decreasing acidity.

The exergonic character of the transfer reactions described in Equations 8–11 is largely driven by proton exchange from the organic to the aqueous phase. Although the aqueous medium is strongly acidic, the calculated free energies indicate that proton transfer remains a major stabilizing contribution to the overall extraction process. These findings highlight the fundamental role of coupled ligand-exchange and proton-transfer equilibria at the phase boundary. From a process-design perspective, the development of tailored extractants capable of modulating the aqueous hydrolysis of Zr­(IV) and Hf­(IV), while preserving efficient coordination in the organic phase, emerges as a key strategy for enhancing selectivity. Such control over hydrolysis and interfacial proton exchange may enable more efficient and environmentally sustainable Hf­(IV)/Zr­(IV) separation processes.

Experiments were also carried out to evaluate the efficiency of D2EHPA for Hf­(IV)/Zr­(IV) separation using stock solutions derived directly from sulfuric liquors obtained from mineral sources, thereby reproducing realistic processing conditions. The presence of potentially competing impurity ions significantly affected the extraction process, reducing the separation factor to approximately 1.5, in contrast to the much larger values typically reported in studies employing analytical-grade reagents. The experimental results further demonstrate that aging of the feed solution substantially modifies metal speciation, increasing zirconium extraction and promoting pronounced sulfate coextraction. These findings confirm that sulfate-coordinated species play a decisive role in governing extraction behavior under industrially relevant conditions.

Overall, the combined experimental–theoretical approach demonstrates that Hf­(IV)/Zr­(IV) separation in sulfate media is governed by tightly coupled hydrolysis, sulfate coordination, and proton-exchange equilibria. The present results highlight the importance of controlling feed composition, aging time, and sulfate concentration in order to optimize extraction efficiency and selectivity in industrial hydrometallurgical processing of nuclear-grade zirconium.

## Supplementary Material



## Notes

Elemental composition of the zirconium concentrate supplied by INB and the sulfuric acid feed liquor; optimized structures of the calculated species; calculated reaction Gibbs free energies; and selected bond distances of the optimized species (PDF)
