# Supplementary material for: Mechanistic Insights into Hf(IV)/Zr(IV) Extraction by D2EHPA in Sulfate Media: A DFT Study Supported by Experiments with Industrial Sulfate Liquors
Source: ACS Omega. 2026 Aug 19;11(34):51721–30. doi: 10.1021/acsomega.6c06044 (PMC13625078; doi:10.1021/acsomega.6c06044)
Supplement: Supplementary file 1 [file ao6c06044_si_001.pdf]

SUPPORTING INFORMATION

Mechanistic Insights Hf(IV)/Zr(IV) Extraction by D2EHPA in Sulfate Media: A DFT Study Supported by Experiments with Industrial Sulfate Liquors.

Pedro Augusto de Andrade Novaes[1], Raquel M. Mingote[2], Carlos A. Morais[2], Alexandre Bertoli[3], Hélio Anderson Duarte*[1].

1. Grupo de Pesquisa em Química Inorgânica Teórica – GPQIT, Departamento de Química – ICEx, Universidade Federal de Minas Gerais – UFMG, 31270-901 – Belo Horizonte - MG, Brazil.
2. Centro de Desenvolvimento da Tecnologia Nuclear – CDTN, 31270-901 – Belo Horizonte – MG, Brazil.
3. Departamento de Química Inorgânica, Centro de Tecnologia, Universidade Federal do Rio de Janeiro – UFRJ, 21941-909 – Rio de Janeiro – RJ, Brazil.

*e-mail: duarteh@ufmg.br

Contents

Figures

Figure S1. Optimized geometries of Hf(IV) aqua complexes at the PBE/TZVP level: $[Hf(H_2O)_7]^{4+}$ and $[Hf(H_2O)_8]^{4+}$………………………………………………………….. 4
Figure S2. Optimized structures for (a) Hf(IV) and (b) Zr(IV) hydrolysis reactions (CN=8) calculated at the PBE/def2-TZVP level with CPCM. ……………………………………….. 5
Figure S3. Optimized structures for sulfate coordination reactions of (a) Zr(IV) and (b) Hf(IV) complexes with CN = 8 at the PBE level. …………………………………………………… 7

Tables

Table S1. Elemental composition of the zirconium concentrate supplied by INB. Mineral constituents with contents greater than 0.5% are presented………………………………….. 3
Table S2. Elemental composition of the sulfuric acid feed liquor…………………………… 3
Table S3. Average Zr(IV)/Hf(IV)–H_2O bond distances (Å) for NC = 7–8 complexes, using different XC functionals……………………………………………………………………… 4
Table S4. Gibbs free energies (kcal mol^{-1}) of the complexes with NC=8 and 8 calculated using different XC functionals with the CPCM implicit solvation model, according to Eq. (5). ….. 4
Table S5. Gibbs free energies (kcal mol^{-1}) for the hydrolysis reactions of complexes with NC=7 and 8 calculated using the PBE XC with different basis sets. ………………………… 5
Table S6. Calculated free energies (kcal mol^{-1}) for the hydrolysis reactions of complexes with NC=7 and 8, obtained using the CPCM solvation model and the def2-TZVP basis set…… 6
Table S7 - Calculated complexation free energies (kcal mol^{-1}) for sulfate coordination reactions using different basis sets………………………………………………………… 6
Table S8. Calculated complexation free energies (kcal mol^{-1}) for sulfate coordination reactions at NCs 7 and 8, computed using the CPCM solvation model and the def2-TZVP basis set… 7
Table S9 - Calculated reaction free energies (kcal mol^{-1}) for the liquid–liquid extraction pathways at the aqueous-organic interface, computed at the PBE/CPCM level of theory…… 8
Table S10. Calculated reaction energies (kcal mol^{-1}) for the liquid–liquid extraction pathway at the aqueous/organic interface computed with the CPCM solvation model………………… 8
Table S11. Binding free energy (in kcal mol-1) of the hydrogen-bonded D2EHPA dimer… 8
Table S12. Hf(IV)-H2O bond distances (Å) for NC = 7 and 8…………………………….. 9
Table S13. Zr(IV)-H2O bond distances (Å) for NC = 7 and 8……………………………… 9
Table S14. Hf(IV)--H2O/OH bond distances (Å), hydrolyzed species…………………… 9
Table S15. Zr(IV)--H2O/OH bond distances (Å), hydrolyzed species……………………… 9
Table S16. Hf(IV)--H2O/OH bond distances (Å), hydrolyzed species……………………. 9
Table S17. Zr(IV)-H2O/OH bond distances (Å), hydrolyzed species……………………… 10
Table S18. Hf(IV)--H2O/M=O bond distances (Å), oxo species…………………………...... 10
Table S19. Zr(IV)-H2O/M=O bond distances (Å), oxo species……………………………..10
Table S20. Hf(IV)--H2O/SO4 bond distances (Å), sulfate-coordinated species…………….10
Table S21. Zr(IV)-H2O/SO4 bond distances (Å), sulfate-coordinated species…………......10
Table S22. Hf(IV)-H2O/SO4 bond distances (Å), sulfate-coordinated species…………… 11
Table S23. Zr(IV)-H2O/SO4 bond distances (Å), sulfate-coordinated species……………. 11

Experimental Data

Table S1. Elemental composition of the zirconium concentrate supplied by INB. Mineral constituents with contents greater than 0.5% are presented.

Mineral	Weight (%)
ZrO_2	65.0
HfO_2	1.33
SiO_2	29.0
Al_2O_3	2.22
TiO_2	0.94
BaO	0.58
Fe_2O_3	0.53

Table S2. Elemental composition of the sulfuric acid feed liquor.

Element	Concentration (g L^{-1})
Zr	20.2
Hf	0.428
Al	0.129
Fe	0.060
Si	0.002

Coordination Number Analysis of Aqueous Zr(IV) and Hf(IV) Complexes.

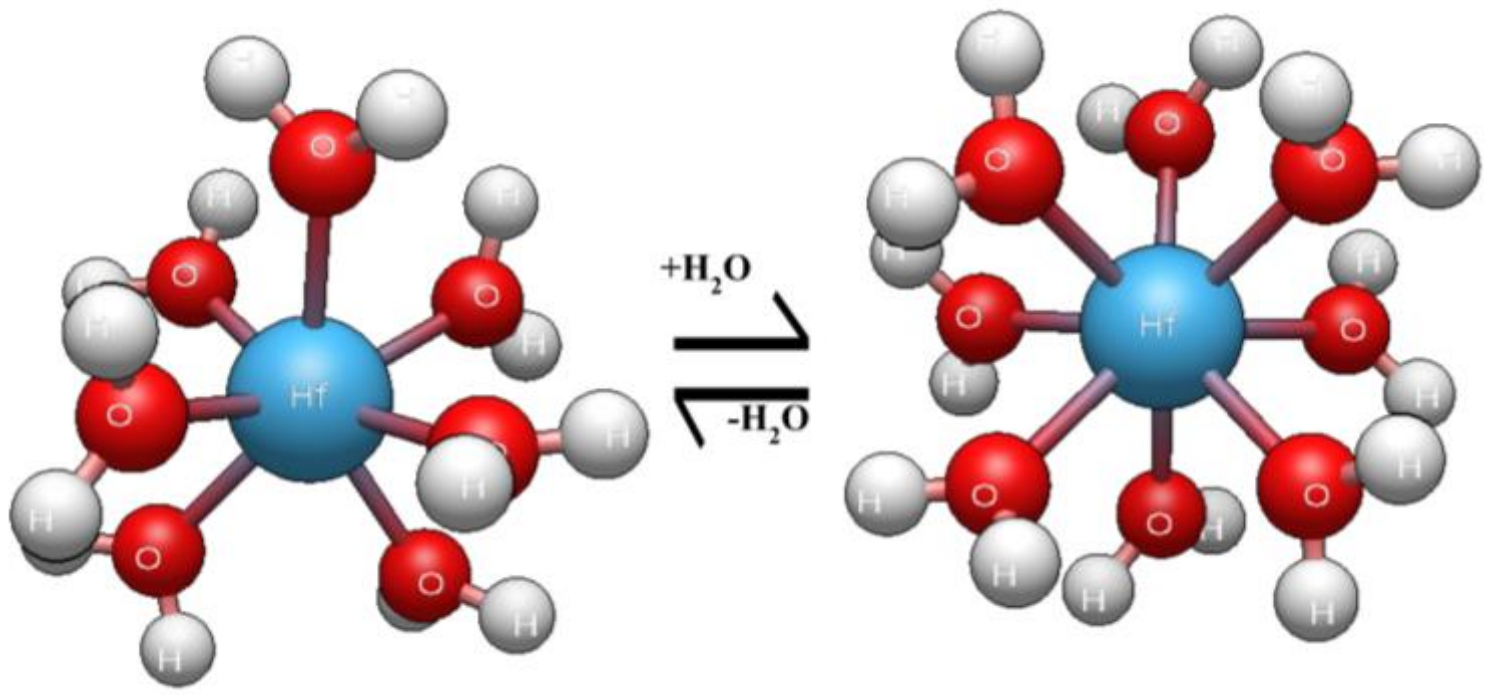

Figure S1. Optimized geometries of Hf(IV) aqua complexes at the PBE/TZVPPD level: $[Hf(H_2O)_7]^{4+}$ and $[Hf(H_2O)_8]^{4+}$.

Table S3. Average Zr(IV)/Hf(IV)–H_2O bond distances (Å) for NC = 7–8 complexes, using different XC functionals.

Metal	PBE		PBE0		PBE-D3	
	Bond Length (Å)					
	NC=7	NC=8	NC=7	NC=8	NC=7	NC=8
**Zr(IV)**	2.17	2.25	2.18	2.19	2.17	2.26
**Hf(IV)**	2.16	2.21	2.15	2.19	2.16	2.20

Table S4. Gibbs free energies (kcal mol^{-1}) of the complexes with NC=8 and 8 calculated using different XC functionals with the CPCM implicit solvation model, according to Eq. (5).

	Def2- TZVP			Def2-TZVPPD*
	PBE	PBE0	PBE-D3	PBE
**Zr(IV)**	-10.5	-11.81	-13.17	-7.5
**Hf(IV)**	-11.2	-11.15	-13.97	-8.1

* ΔE values were calculated at the PBE/def2-TZVPPD level using PBE/def2-TZVP optimized geometries. The Gibbs free energies were then recomputed by combining the single-point electronic energies obtained with the def2-TZVPPD basis set with the thermal corrections and CPCM solvation contributions calculated at the PBE/def2-TZVP level.

Hydrolysis of the Zr(IV) and Hf(IV).

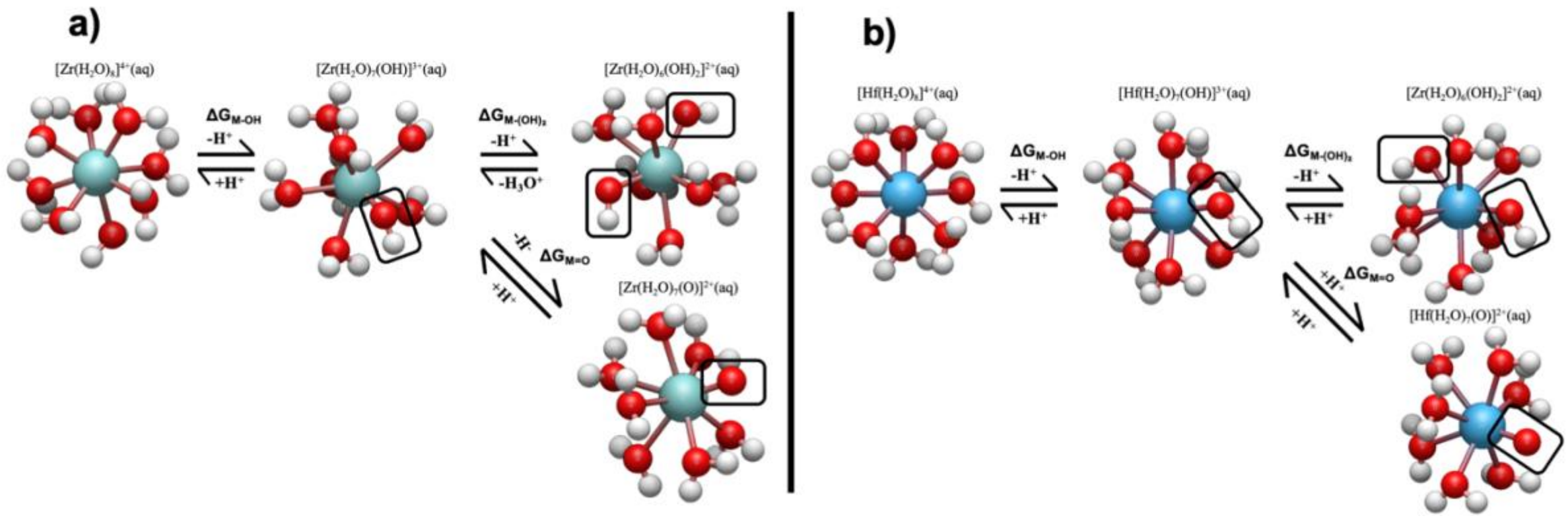

Figure S2. Optimized structures for (a) Hf(IV) and (b) Zr(IV) hydrolysis reactions (CN=8) calculated at the PBE/def2-TZVP level with CPCM.

Table S5. Gibbs free energies (kcal mol⁻¹) for the hydrolysis reactions of complexes with NC=7 and 8 calculated using the PBE XC with different basis sets.

		**Def2-TZVP**			**Def2-TZVPPD***		
**Metal**	**NC**	**1st Stage**	**2nd Stage**		**1st Stage**	**2nd Stage**	
		$\Delta G_{M\text{-}OH}$	$\Delta G_{M\text{-}(OH)2}$	$\Delta G_{M=O}$	$\Delta G_{M\text{-}OH}$	$\Delta G_{M\text{-}(OH)2}$	$\Delta G_{M=O}$
**Zr(IV)**	7	-32.6	-12.2	-1.1	-31.6	-13.0	-0.5
	8	-19.7	-4.7	-2.2	-19.5	-4.5	-2.0
**Hf(IV)**	7	-31.1	-7.9	6.5	-30.2	-8.1	6.6
	8	-17.0	6.5	5.9	-17.0	-3.1	6.5

* ΔE values were calculated at the PBE/def2-TZVPPD level using PBE/def2-TZVP optimized geometries. The Gibbs free energies were then recomputed by combining the single-point electronic energies obtained with the def2-TZVPPD basis set with the thermal corrections and CPCM solvation contributions calculated at the PBE/def2-TZVP level.

Table S6. Calculated free energies (kcal mol^{-1}) for the hydrolysis reactions of complexes with NC=7 and 8, obtained using the CPCM solvation model and the def2-TZVP basis set.

		PBE0 (kcal mol^{-1})			PBE-D3 (kcal mol^{-1})		
**Metal**	**NC**	**1st Stage**	**2nd Stage**		**1st Stage**	**2nd Stage**	
		ΔG_{M-OH}	$\Delta G_{M-(OH)2}$	$\Delta G_{M=O}$	ΔG_{M-OH}	$\Delta G_{M-(OH)2}$	$\Delta G_{M=O}$
**Zr(IV)**	7	-30.04	-9.46	3.03	-32.23	-10.93	0.33
	8	-17.91	-0.13	4.92	-18.76	-4.01	-0.07
**Hf(IV)**	7	-28.50	-5.49	11.11	-30.16	-8.07	7.37
	8	-15.72	0.22	13.34	-15.66	-2.09	7.43

Table S7 - Calculated complexation free energies (kcal mol^{-1}) for sulfate coordination reactions using different basis sets and PBE XC.

	**Def2-TZVP**		**Def2-TZVPPD***	
**Metal**	**CN = 7**	**CN = 8**	**CN = 7**	**CN = 8**
**Zr(IV)**	-69.6	-59.8	-58.9	-50.0
**Hf(IV)**	-71.0	-57.2	-60.5	-47.7

*ΔE values were calculated at the PBE/def2-TZVPPD level using PBE/def2-TZVP optimized geometries. The Gibbs free energies were then recomputed by combining the single-point electronic energies obtained with the def2-TZVPPD basis set with the thermal corrections and CPCM solvation contributions calculated at the PBE/def2-TZVP level.

Sulfate coordination to the hydrolyzed Zr(IV) and Hf(IV) species in aqueous solution.

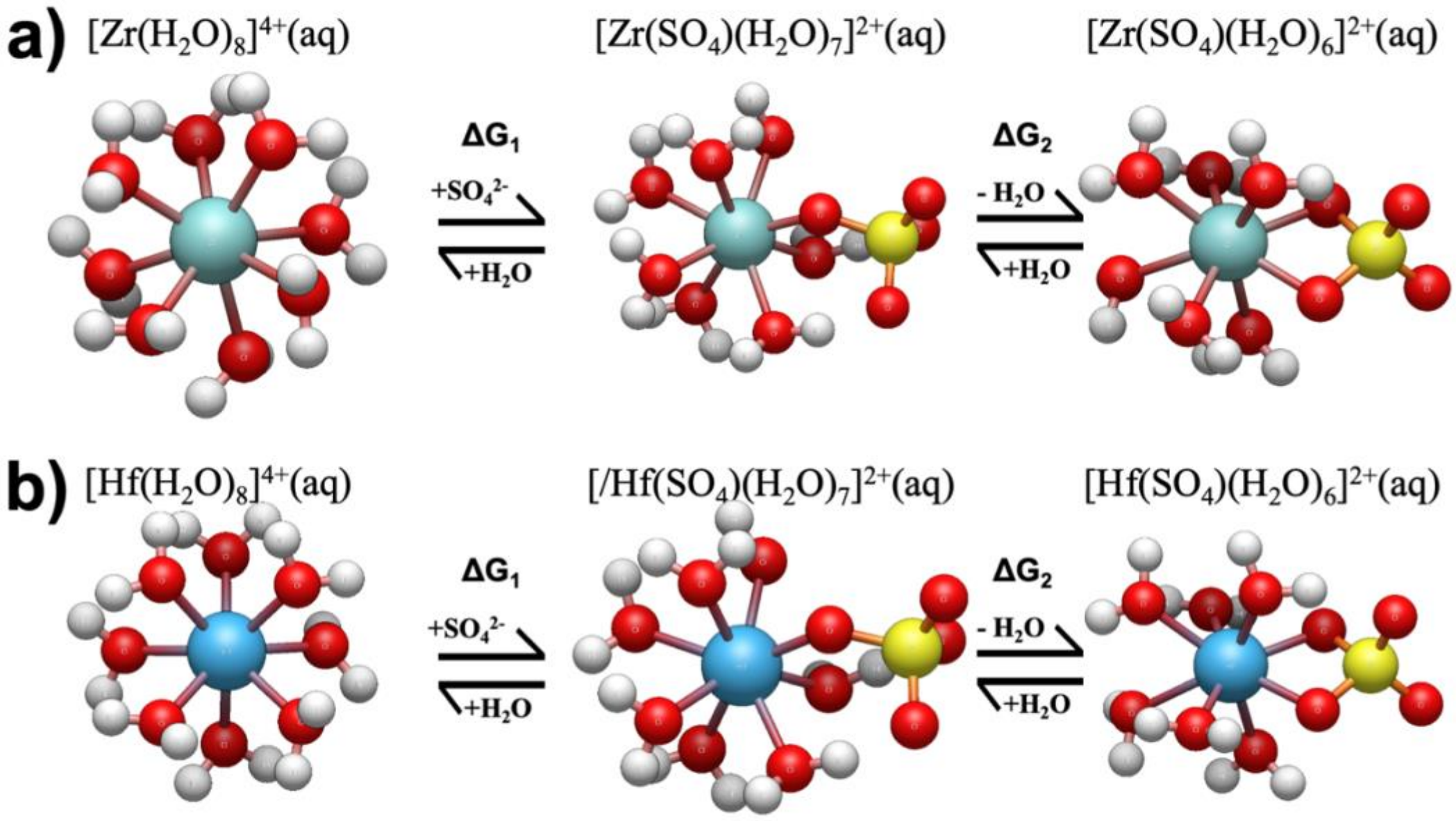

Figure S3. Optimized structures for sulfate coordination reactions of (a) Zr(IV) and (b) Hf(IV) complexes with CN = 8 at the PBE level.

Table S8. Calculated complexation free energies (kcal mol^{-1}) for sulfate coordination reactions at NCs 7 and 8, computed using the CPCM solvation model and the def2-TZVP basis set.

	**PBE0 (kcal mol^{-1})**				**PBE-D3 (kcal mol^{-1})**			
	**CN = 7**		**CN = 8**		**CN = 7**		**CN = 8**	
	**ΔG_1**	**ΔG_2**	**ΔG_1**	**ΔG_2**	**ΔG_1**	**ΔG_2**	**ΔG_1**	**ΔG_2**
**Zr(IV)**	-63.77	-5.27	-51.67	-4.73	-73.10	1.29	-60.91	-1.44
**Hf(IV)**	-77.51	9.90	-50.99	-4.09	-71.66	-0.56	-58.67	-0.79

Liquid-liquid extraction mechanism of the Zr and Hf with D2EHPA extractant.

Table S9 - Calculated reaction free energies (kcal mol⁻¹) for the liquid–liquid extraction pathways at the aqueous-organic interface, computed at the PBE/CPCM level of theory.

	**Def2-TZVP**				**Def2-TZVPPD***			
**M**	**Zr(IV)**		**Hf(IV)**		**Zr(IV)**		**Hf(IV)**	
**CN**	**7**	**8**	**7**	**8**	**7**	**8**	**7**	**8**
ΔG_{I}	-17.9	-13.3	-18.4	-11.5	-24.9	-19.9	-25.4	-18.1
ΔG_{II}	-16.6	-6.8	-10.4	-4.9	-20.1	-10.0	-14.2	-8.4
ΔG_{III}	-7.2	-9.1	-10.3	-7.3	-13.7	-16.3	-17.7	-14.0
ΔG_{IV}	1.0	5.9	2.8	5.6	-2.0	2.0	-1.6	1.8

* ΔE values were calculated at the PBE/def2-TZVPPD level using PBE/def2-TZVP optimized geometries. The Gibbs free energies were then recomputed by combining the single-point electronic energies obtained with the def2-TZVPPD basis set with the thermal corrections and CPCM solvation contributions calculated at the PBE/def2-TZVP level.

Table S10. Calculated reaction energies (kcal mol⁻¹) for the liquid–liquid extraction pathway at the aqueous/organic interface computed with the CPCM solvation model.

	**PBE0 (kcal mol⁻¹)**				**PBE-D3 (kcal mol⁻¹)**			
	**Zr(IV)**		**Hf(IV)**		**Zr(IV)**		**Hf(IV)**	
**CN**	**7**	**8**	**7**	**8**	**7**	**8**	**7**	**8**
ΔG_{I}	-37.44	-33.41	-38.59	-31.73	-33.6	-33.5	-32.6	-31.5
ΔG_{II}	-22.57	-15.65	-20.09	-13.71	-22.9	-10.9	-16.6	-9.0
ΔG_{III}	-27.23	-29.62	-28.84	-25.89	-26.6	-31.7	-28.8	-26.2
ΔG_{IV}	-7.44	-1.85	-5.24	-1.37	-5.1	0.8	-1.6	0.4

Table S11. Binding free energy (in kcal mol⁻¹) of the hydrogen-bonded D2EHPA dimer.

Method	ΔE
Non-BSSE corrected	-21.88
BSSE[1] corrected	-20.84

[1]Boys, S. F.; Bernardi, F. *Mol. Phys.* 1970, *19*, 553.

Selected bonding distances

Table S12. Hf(IV)-H_2O bond distances (Å) for NC = 7 and 8.

System	XC	OH_2-1	OH_2-2	OH_2-3	OH_2-4	OH_2-5	OH_2-6	OH_2-7	OH_2-8	Mean
Hf NC7	PBE	2.183	2.175	2.171	2.159	2.159	2.158	2.153	--	2.16
Hf NC7	PBE0	2.169	2.157	2.156	2.145	2.145	2.144	2.138	--	2.15
Hf NC7	PBE-D3	2.181	2.172	2.169	2.156	2.156	2.156	2.150	--	2.16
Hf NC8	PBE	2.217	2.213	2.212	2.209	2.203	2.201	2.200	2.200	2.21
Hf NC8	PBE0	2.194	2.193	2.192	2.191	2.190	2.189	2.188	2.187	2.19
Hf NC8	PBE-D3	2.212	2.211	2.204	2.204	2.203	2.202	2.197	2.196	2.20

Table S13. Zr(IV)-H_2O bond distances (Å) for NC = 7 and 8.

System	XC	OH_2-1	OH_2-2	OH_2-3	OH_2-4	OH_2-5	OH_2-6	OH_2-7	OH_2-8	Mean
Zr NC7	PBE	2.180	2.179	2.174	2.172	2.167	2.156	2.150	--	2.17
Zr NC7	PBE0	2.208	2.192	2.192	2.181	2.179	2.173	2.171	--	2.18
Zr NC7	PBE-D3	2.182	2.174	2.169	2.167	2.167	2.152	2.147	--	2.17
Zr NC8	PBE	2.263	2.262	2.258	2.252	2.250	2.246	2.243	2.235	2.25
Zr NC8	PBE0	2.195	2.194	2.193	2.192	2.192	2.191	2.191	2.189	2.19
Zr NC8	PBE-D3	2.268	2.261	2.260	2.256	2.255	2.242	2.232	2.228	2.26

Table S14. Hf(IV)--H_2O/OH bond distances (Å), hydrolyzed species.

System	XC	OH_2-1	OH_2-2	OH_2-3	OH_2-4	OH_2-5	OH_2-6	OH_2-7	OH
Hf NC7	PBE	2.235	2.234	2.231	2.230	2.223	2.214	--	1.879
Hf NC7	PBE0	2.244	2.236	2.235	2.225	2.219	2.213	--	1.879
Hf NC7	PBE-D3	2.252	2.238	2.235	2.229	2.215	2.208	--	1.879
Hf NC8	PBE	2.386	2.282	2.281	2.279	2.245	2.238	2.232	1.926
Hf NC8	PBE0	2.337	2.284	2.242	2.238	2.235	2.219	2.205	1.930
Hf NC8	PBE-D3	2.379	2.278	2.277	2.276	2.242	2.235	2.228	1.927

Table S15. Zr(IV)--H_2O/OH bond distances (Å), hydrolyzed species.

System	XC	OH_2-1	OH_2-2	OH_2-3	OH_2-4	OH_2-5	OH_2-6	OH_2-7	OH
Zr NC7	PBE	2.264	2.255	2.248	2.242	2.234	2.233	--	1.866
Zr NC7	PBE0	2.280	2.251	2.241	2.241	2.228	2.227	--	1.912
Zr NC7	PBE-D3	2.282	2.275	2.240	2.221	2.210	2.206	--	1.867
Zr NC8	PBE	2.387	2.319	2.277	2.269	2.261	2.248	2.235	1.924
Zr NC8	PBE0	2.332	2.294	2.285	2.279	2.258	2.251	2.250	1.955
Zr NC8	PBE-D3	2.368	2.314	2.311	2.306	2.290	2.280	2.267	1.967

Table S16. Hf(IV)--H_2O/OH bond distances (Å), hydrolyzed species.

System	XC	OH_2-1	OH_2-2	OH_2-3	OH_2-4	OH_2-5	OH_2-6	OH-1	OH-2
Hf NC7	PBE	2.295	2.292	2.282	2.269	2.258	--	1.954	1.954
Hf NC7	PBE0	2.269	2.266	2.256	2.247	2.237	--	1.941	1.941
Hf NC7	PBE-D3	2.293	2.286	2.285	2.267	2.255	--	1.955	1.955
Hf NC8	PBE	2.360	2.343	2.333	2.332	2.264	2.258	2.028	2.018
Hf NC8	PBE0	2.352	2.308	2.304	2.300	2.243	2.238	2.013	2.006
Hf NC8	PBE-D3	2.354	2.338	2.329	2.328	2.260	2.254	2.028	2.018

Table S17. Zr(IV)-H_2O/OH bond distances (Å), hydrolyzed species.

System	XC	OH_2-1	OH_2-2	OH_2-3	OH_2-4	OH_2-5	OH_2-6	OH 1	OH 2
Zr NC7	PBE-D3	2.288	2.275	2.261	2.255	2.245	--	1.999	1.970
Zr NC7	PBE	2.371	2.300	2.284	2.283	2.280	--	1.965	1.918
Zr NC7	PBE0	2.284	2.280	2.267	2.266	2.258	--	2.025	1.979
Zr NC8	PBE-D3	2.379	2.352	2.345	2.324	2.273	2.271	2.017	2.012
Zr NC8	PBE0	2.420	2.326	2.316	2.303	2.268	2.263	2.028	2.023
Zr NC8	PBE	2.389	2.358	2.353	2.327	2.278	2.275	2.017	2.011

Table S18. Hf(IV)--H_2O/M=O bond distances (Å), oxo species.

System	XC	OH_2-1	OH_2-2	OH_2-3	OH_2-4	OH_2-5	OH_2-6	OH_2-7	M=O
Hf NC7	PBE	2.414	2.303	2.281	2.280	2.279	2.271	--	1.791
Hf NC7	PBE0	2.387	2.274	2.272	2.258	2.254	2.253	--	1.785
Hf NC7	PBE-D3	2.408	2.291	2.289	2.277	2.272	2.272	--	1.799
Hf NC8	PBE	3.925	2.437	2.304	2.297	2.297	2.286	2.247	1.801
Hf NC8	PBE0	2.569	2.383	2.304	2.300	2.287	2.265	2.265	1.795
Hf NC8	PBE-D3	2.617	2.437	2.330	2.321	2.304	2.280	2.279	1.827

Table S19. Zr(IV)-H_2O/M=O bond distances (Å), oxo species.

System	XC	OH_2-1	OH_2-2	OH_2-3	OH_2-4	OH_2-5	OH_2-6	OH_2-7	M=O
Zr NC7	PBE	2.445	2.317	2.303	2.294	2.291	2.285	--	1.776
Zr NC7	PBE0	2.478	2.309	2.305	2.289	2.284	2.284	--	1.784
Zr NC7	PBE-D3	2.531	2.325	2.321	2.316	2.314	2.308	--	1.795
Zr NC8	PBE	3.942	2.497	2.343	2.332	2.331	2.319	2.293	1.799
Zr NC8	PBE0	2.595	2.407	2.323	2.319	2.304	2.285	2.284	1.765
Zr NC8	PBE-D3	4.009	2.462	2.313	2.312	2.307	2.302	2.253	1.779

Table S20. Hf(IV)--H_2O/SO_4 bond distances (Å), sulfate-coordinated species.

System	XC	OH_2-1	OH_2-2	OH_2-3	OH_2-4	OH_2-5	OH_2-6	OH_2-7	SO_4-1
Hf NC7	PBE	2.229	2.220	2.215	2.202	2.160	2.158	--	2.038
Hf NC7	PBE0	2.205	2.194	2.191	2.176	2.143	2.141	--	2.034
Hf NC7	PBE-D3	2.232	2.214	2.212	2.210	2.193	2.188	--	2.003
Hf NC8	PBE	2.294	2.275	2.268	2.261	2.248	2.241	2.211	2.066
Hf NC8	PBE0	2.267	2.247	2.243	2.230	2.221	2.211	2.196	2.059
Hf NC8	PBE-D3	2.287	2.271	2.264	2.258	2.244	2.239	2.206	2.066

Table S21. Zr(IV)-H_2O/SO_4 bond distances (Å), sulfate-coordinated species.

System	XC	OH_2-1	OH_2-2	OH_2-3	OH_2-4	OH_2-5	OH_2-6	OH_2-7	SO_4-1
Zr NC7	PBE	2.267	2.252	2.241	2.241	2.197	2.189	--	2.031
Zr NC7	PBE0	2.255	2.223	2.223	2.221	2.210	2.140	--	1.985
Zr NC7	PBE-D3	2.265	2.248	2.238	2.237	2.195	2.188	--	2.030
Zr NC8	PBE	2.331	2.309	2.291	2.285	2.276	2.262	2.229	2.054
Zr NC8	PBE0	2.290	2.273	2.271	2.259	2.255	2.233	2.208	2.039
Zr NC8	PBE-D3	2.325	2.303	2.287	2.283	2.272	2.258	2.225	2.055

Table S22. Hf(IV)-H_2O/SO_4 bond distances (Å), sulfate-coordinated species.

System	XC	OH_2-1	OH_2-2	OH_2-3	OH_2-4	OH_2-5	OH_2-6	SO_4-1	SO_4-2
Hf NC7	PBE	2.228	2.201	2.199	2.180	2.179	--	2.098	2.085
Hf NC7	PBE0	2.205	2.180	2.179	2.161	2.161	--	2.084	2.077
Hf NC7	PBE-D3	2.222	2.197	2.195	2.177	2.177	--	2.096	2.084
Hf NC8	PBE	2.302	2.271	2.250	2.241	2.202	2.200	2.159	2.129
Hf NC8	PBE0	2.276	2.238	2.233	2.214	2.185	2.182	2.142	2.118
Hf NC8	PBE-D3	2.296	2.267	2.246	2.238	2.198	2.197	2.158	2.128

Table S23. Zr(IV)-H_2O/SO_4 bond distances (Å), sulfate-coordinated species.

System	XC	OH_2-1	OH_2-2	OH_2-3	OH_2-4	OH_2-5	OH_2-6	SO_4-1	SO_4-2
Zr NC7	PBE	2.245	2.206	2.206	2.183	2.182	--	2.088	2.082
Zr NC7	PBE0	2.222	2.184	2.184	2.165	2.163	--	2.074	2.069
Zr NC7	PBE-D3	2.229	2.208	2.202	2.190	2.188	--	2.090	2.081
Zr NC8	PBE	2.313	2.274	2.261	2.253	2.209	2.208	2.148	2.128
Zr NC8	PBE0	2.279	2.245	2.240	2.226	2.191	2.188	2.133	2.113
Zr NC8	PBE-D3	2.304	2.276	2.258	2.251	2.205	2.205	2.152	2.120
